# Leasing-Based Performance Analysis in Energy Harvesting Cognitive Radio Networks

**DOI:** 10.3390/s16030305

**Published:** 2016-02-27

**Authors:** Fanzi Zeng, Jisheng Xu

**Affiliations:** College of Information Science and Engineering, Hunan University, Changsha, 410012, China; zengfanzi@126.com

**Keywords:** cognitive radio, spectrum leasing, energy harvesting, optimal strategy, energy efficiency ratio

## Abstract

In this paper, we consider an energy harvesting cognitive radio network (CRN), where both of primary user (PU) and secondary user (SU) are operating in time slotted mode, and the SU powered exclusively by the energy harvested from the radio signal of the PU. The SU can only perform either energy harvesting or data transmission due to the hardware limitation. In this case, the entire time-slot is segmented into two non-overlapping fractions. During the first sub-timeslot, the SU can harvest energy from the ambient radio signal when the PU is transmitting. In order to obtain more revenue, the PU leases a portion of its time to SU, while the SU can transmit its own data by using the harvested energy. According to convex optimization, we get the optimal leasing time to maximize the SU’s throughput while guaranteeing the quality of service (QoS) of PU. To evaluate the performance of our proposed spectrum leasing scheme, we compare the utility of PU and the energy efficiency ratio of the entire networks in our framework with the conventional strategies respectively. The numerical simulation results prove the superiority of our proposed spectrum leasing scheme.

## 1. Introduction

With the rapid development and extensive application of wireless communication technology, limited spectrum resource and inflexible spectrum assignment policy bring about poor spectrum utilization currently. Cognitive radio technology that allows smart spectrum sharing between licensed (primary) users and unlicensed (secondary) users emerged in 1999 which is regarded as a promising technology for improving spectrum utilization [1].

There are two kinds of spectrum sharing modes between the PU and the SU. One is spectrum sensing that the unlicensed secondary users can access the channels that are not currently occupied by the licensed primary users through spectrum sensing to avoid harmful interference on the primary user(PU) for data transmission. Another is spectrum leasing which is adopted in this paper, specifically, the PU is aware of existence of the SU and it can gain some revenue by leasing parts of spectrum to the SU. There have been many literatures about research in the lease spectrum. In [2], the authors proposed the "cooperative cognitive radio networks" in which PUs rent a fraction of the owned spectrum to SUs in exchange for cooperative relaying and model the spectrum leasing strategies as a Stackelberg game. Similar scenario and model take place in [3], where a primary link has the possibility to lease the licensed spectrum to an ad hoc network of secondary users in return for assistance in the form of distributed space-time coding. The authors in [4] investigated multi-access protocols allowing simultaneous transmission of relaying and secondary database on dirty-paper coding (DPC) with considering cooperation between PUs and SUs, which introduces a trade-off between PU and SU performance combining with opportunistic relay selection. In [5], the authors studied an overlay spectrum sharing scheme where the SU leases half of the time-slot from the PU at the cost of relaying the PU’s data transmission, they proposed the SU’s antenna combining weights as well as power allocation scheme to encounter a certain error or rate design standard for both PU and SU. In [6], considered the problem of two wireless networks operating on the same frequency band. Pairs within a given network cooperate to schedule transmissions, but between networks there is competition for spectrum.

In recent years, the problem about energy consumption has become increasingly severe; this urgently calls for solutions to improve energy efficiency [7]. Apart from energy-efficient wireless communication technologies, energy harvesting communication systems powered either largely or exclusively by renewable sources are regarded as another useful alternative that can efficiently relieve energy-deficiency and have become more attractive [8]. Compared to traditional battery-powered communication systems, energy harvesting could potentially provide infinite energy supply from the environment in a much easier and safer way for conventional energy-constrained wireless communication systems, such as wireless sensor networks (WSNs). Pappas *et al.* studied the maximum stable throughput region for a simple cognitive radio network when the primary transmitter harvests ambient energy but the secondary transmitter is plugged into a reliable power supply [9]. Lee *et al.* analyzed the transmission probability of secondary transmitters and the resulting spatial throughput of the secondary network when the secondary transmitters harvest ambient RF energy from transmissions by nearby active primary transmitters, while opportunistically accessing the spectrum licensed to the primary network [10]. Energy harvesting is also regarded as a promising approach for powering relay node [11,12,13,14,15]. In [11], the authors derived the optimal switching rule between the information decoding mode and the energy harvesting mode to minimize the outage probability. In [12], the authors formulated the optimal sensing and access policies as a Markov decision process and derived the optimal policy by the probability of PU’s presence and the amount of stored energy. In [13], the authors considered a cognitive radio sensor network powered by RF energy harvesting and formulated the optimal mode selection (“access” or “harvest”) policy as a partially observable Markov decision process (POMDP). In [15], spatial spectrum reused in CR networks with energy harvesting was investigated based on a stochastic-geometry model where the primary and secondary transmitters are modeled as independent homogeneous Poisson point processes (HPPPs). In [14], the authors provided an optimal spectrum sensing policy for an energy-harvesting CR that can maximize the expected total throughput under energy causality and collision constraints.

In [16], a multi-antenna model was proposed where the receiver can only harvest energy from the transmitter by wireless energy transfer to serve for its data transmission. In [17], the authors discussed an efficient resource-allocation strategy for a multiuser multiple-input-multiple-output rate less coded cognitive radio network with quality-of-service provisioning. The authors of [18] designed an efficient MAC protocol with selective grouping and cooperative sensing in CR networks. Generally, due to the dynamic and random feature of energy in natural circumstance, we consider merely a scenario that wireless communication device collects energy from ambient wireless signal. Compared with these works, the salient feature of this paper is that, by considering the aforementioned trade-offs between energy harvesting and secondary transmission, we investigate the optimal secondary transmission duration time that maximizes the SU’s achievable throughput and further present energy efficiency ratio of the entire network based on the optimal closed-form solutions.

Besides, we introduce an important concept: utility. In economics, utility is a measure of preference over some set of goods and services. The concept is an important underpinning of rational choice theory in economics and game theory, because it represents consumers’ satisfaction. A good is something that satisfies human wants. Since one cannot directly measure benefit, satisfaction or happiness from a good or service, economists have devised ways of representing and measuring utility in terms of economic choices that can be measured. Economists have attempted to improve highly-abstract methods of comparing utilities by observing and calculating economic choices. In the simplest sense, economists consider utility to be revealed by people’s willingness to pay different amounts of money for different goods. There are some papers which discussed utility-based resource allocation in wireless networks. In [19], the authors presented a time-aware admission control and resource allocation scheme in wireless networks in the context of a future generation cellular network. In [20], the authors studied utility-based resource allocation for soft QoS traffic in infrastructure-based wireless networks.

To achieve the access chance, the SU should make a payment for the PU, which is proportional to the access time. For the PU, the target is to maximize its utility which depends on both its transmission capacity and revenue obtained from the SU. For the SU, the target is to decide how much it should pay for the PU so as to achieve its maximal transmission capacity.

In a word, this paper proposes a spectrum leasing scheme in cognitive radio network with energy harvesting. The SU has equipment which can collect energy from the ambient environment. We assume the SU is powered by harvesting energy and exhausts all the harvesting energy in each time slot for the sake of transmitting data at the highest power. In order to obtain higher energy efficiency of the entire network, the PU prefers to allocate a portion of its spectrum resource to the SU. We derive the optimal spectrum leasing ratio in the following sections. To the best of our knowledge, this work is the first to explore the optimal spectrum leasing ratio in cognitive radio network with energy harvesting, aiming at maximize energy efficiency ratio of the entire network.

The main contributions of this paper are summarized as follows:

- By proving the concavity of the achievable throughput maximization problem in our proposed spectrum leasing model, we derive the closed-form solution.
- We get an improved energy efficiency scheme. Despite reduction of the throughput, the PU can lease its spectrum to SU and allow it to utilize the harvest energy rationally, so as to improve the energy efficiency of the whole system.
- We numerically analyze the impact of various system parameters. The numerical results are consistent with the theoretical analysis.

The rest of the paper is organized as follows. In Section 2, we give a detailed description of the system model and assumptions. Problem formulations are presented in Section 3. We discuss throughput analysis and EER of the model in Section 4. In Section 5, we present and analyze the numerical results. Finally, the conclusion is in Section 6.

## 2. System Model and Assumptions

**Figure 1:**
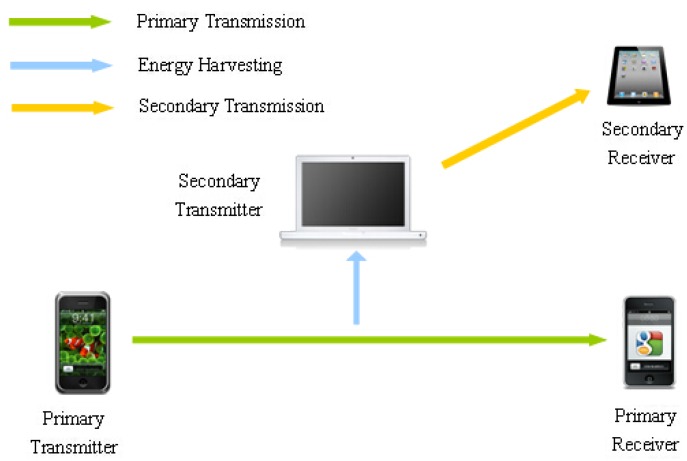
Timeslot structure between secondary user (SU) and primary user (PU) in cognitive radio (CR) systems.

**Figure 2:**
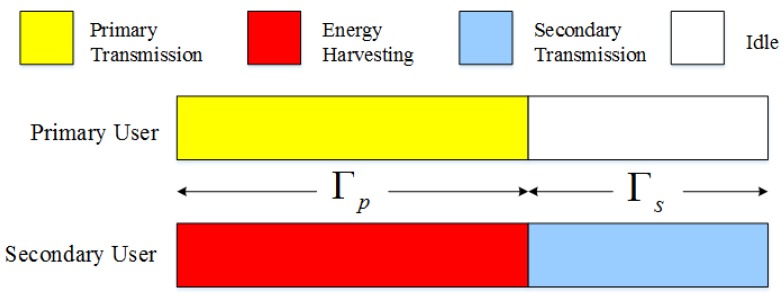
CR systems with energy harvesting between PU and SU.

We assume an energy harvesting cognitive radio network which consists of a PU link pair and a SU link pair as the Figure 1 depicts. Both of them operate in time-slotted mode, as shown in Figure 2. The PU would like to lease a portion of its timeslot to the SU for some revenue only when its utility can be guaranteed.

PU has the priority of using licensed channel and transmits data to its receiver whenever it has data to send. PU has a certain number of data stored in its buffer. In each time-slot, PU uses licensed channel to transmit its data. After the transmission of all the PU’s data, PU turns to be silent and the channel is vacated.

By comparison, SU has no licensed spectrum, to avoid interference on PU ,it has to transmit its data only when the licensed channel is unused by PU. Besides, we assume a self-powered cognitive radio network with a single SU working in slotted mode, the SU has no fixed power supplies (e.g., batteries) and gets power from the ambient radio signal by energy harvester. We consider that SU works in a saving-transmitting way because of the duplex-constrained hardware [8,13]. In each time-slot, the PU uses a fraction of time-slot to transmit data, at the same time, the SU harvests energy exclusively. The rest fraction is used for SU’s data transmission, but SU must pay some remuneration as dividend to the PU.

In such a cognitive radio network, the SU can transmit its data only when the licensed channel is unused by PU so as to avoid interference on the primary receiver. The saving-transmitting structure is shown in Figure 2. In this section, we elaborate the time-slot structure.

The data transmissions are divided into frames with duration of one unit time, and each frame is further partitioned into two fractions for primary usage and secondary usage. The primary user data transmission period, with the duration of $\Gamma_{p}$, $\left(0\leq \Gamma_{p}\leq 1\right)$, is dedicated for the data transmission of primary users. The secondary data transmission period, with the duration of $\Gamma_{s}$ $\left(0\leq \Gamma_{s}\leq 1,\Gamma_{p}+\Gamma_{s}=1\right)$, is dedicated for the data transmission of secondary users, as shown in Figure 2. Which can be detailed as follows:

(1) During time interval $\left(0,\Gamma_{p}\right]$, primary user accesses channel to transmit its data, in the meantime, the SU harvests energy from ambient signal. SU’s harvested energy amounts to $X\Gamma_{p}$, where *X* denotes SU’s energy harvesting rate (average energy harvested in unit time). Meanwhile, the PU completes $R_{p}\Gamma_{p}$ data transmitting, where $R_{p}=\log\left(1+SNR_{p}\right)$.

(2) During time interval $\left(\Gamma_{p},1\right]$, PU’s transmission is finished and the licensed channel is vacated, then the PU leases the vacated channel to SU which can transmit in the remaining time. In return, the SU gives some remuneration to the PU. Then SU starts to transmit its own data. The PU keeps silent and $X\Gamma_{p}$ of energy harvests for secondary transmission. The SU’s signal to noise ratio(SNR) is given as follows, $SNR_{s}=\frac{\Gamma_{p}Xh}{\Gamma_{s}N_{0}}$ where $N_{0}$ is the power of additive white Gaussian noise, *h* denotes the channel gain. The channel model is the foundation for the calculation of transmission rate.

## 3. Problem Formulation

We consider that the PU and the SU co-use the channel, but the PU has priority of accessing the channel and can allocate the access time to the SU. Because the SU can harvest energy from the ambient radio environment, we can use less energy consumption to get better spectrum utilization. But as for getting the optimal allocation policy to have optimal spectrum and energy utilization, there is still a challenge for us.

Transmission time for primary user has an influence on both energy harvesting and data transmitting of the second user. The longer the transmission time of primary user, the more energy the second user can harvest, vice versa. So our main purpose is to find an appropriate transmission time of primary user so as to maximize utility of the primary network.

### 3.1. The Utility Function of Primary Network

**Figure 3:**
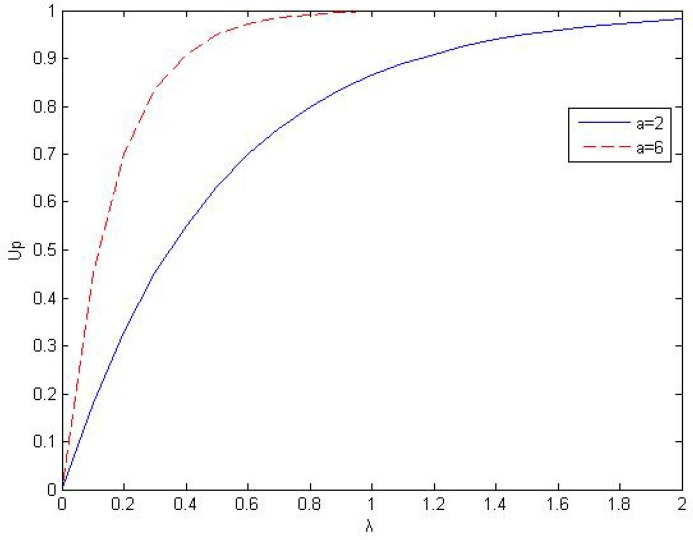
The satisfactory function of primary users $U_{d}$ *versus* *λ*.

The utility function of the primary network consists of two elements: the utility associated with the capacity of primary user, and the revenue gained from secondary user by spectrum renting,

(1) $$U_{p}=\omega_{p}U_{d}+U_{r}.$$

where $U_{r}$ is the revenue gained from secondary network which is defined by $U_{r}=c\Gamma_{s}$, $U_{d}$ is the satisfactory of primary user, $\omega_{p}$ is the equivalent revenue per unit satisfactory conduced to the total utility. The satisfactory of the primary users with respect to their capacity, *i.e.*, $U_{d}$, is defined as follows:

(2) $$U_{d}=1-e^{-\alpha \lambda }.$$

where *α* is the satisfactory factor of the PU(α>1), *λ* is the achieved capacity *versus* its reference capacity, referring to the proportional fairness of resource allocation, which is defined as $\lambda =\frac{C_{p}}{Q}$, where $C_{p}=\Gamma_{p}R_{p}$ is the achieved capacity of the PU, *Q* is the traffic demand of PU, $R_{p}$ is the data rate achieved by primary network transmission, Figure 3 shows the curves of the rate satisfactory function *versus* the *λ* with different *α*. In a word, when the transmission rate increases, the satisfactory function value rapidly reaches an asymptotic value. The greater satisfactory factor, the faster growth of satisfactory function value. It is shown that there is a diminishing marginal rate of satisfactory improvement as capacity increases, which follows the law of the diminishing marginal benefit in economics [4].

### 3.2. The Utility Function of the Secondary Network

Firstly, we define the data rate of the secondary user link $R_{s}$ for its own traffic by Shannon theorem,

(3) $$R_{s}=\log\left(1+SNR_{s}\right)=\log\left(1+\frac{Xh\Gamma_{p}}{\Gamma_{s}N_{0}}\right)$$

where $SNR_{s}$ is the SNR of the channel between the SU transmitter and the secondary user receiver.

**Figure 4:**
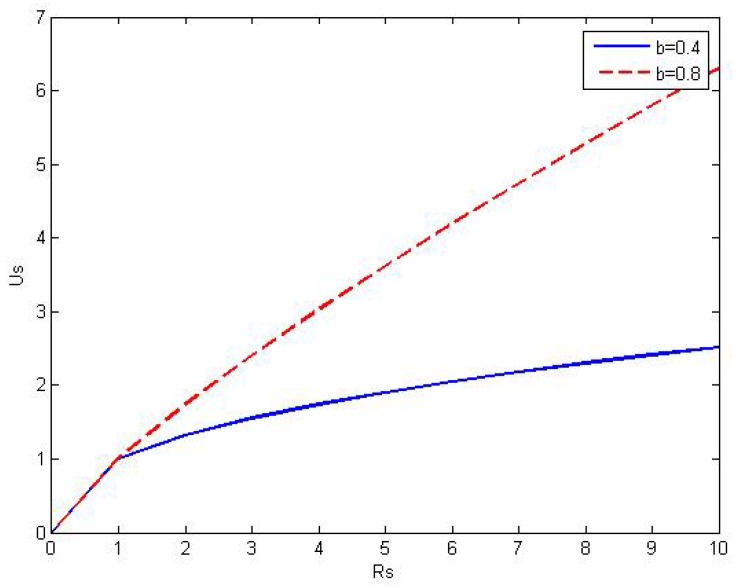
The satisfactory function of secondary user *versus* data rate $R_{s}$.

Then, the utility function of the secondary network is acquired by the utility with respect to secondary users’ capacity minus its payment to the primary network,

(4) $$U_{s}=\omega_{s}{\left(C_{s}\right)}^{b}-c\Gamma_{s}$$

here $C_{s}=\Gamma_{s}R_{s}$ and $\omega_{s}$ is the equivalent revenue in respect of the satisfactory of secondary users conduced to the total utility of the secondary network. The satisfactory function of secondary users is elastic [4], and defined as follows:

(5) $$u_{s}={\left(C_{s}\right)}^{b}$$

where *b* is the satisfactory factor of the SU(0<b<1), Figure 4 illustrates the satisfactory function of the secondary user *versus* its capacity. It is shown that the increment of user’s satisfactory is decreasing as the capacity is increasing, which also follows the law of the diminishing marginal benefit in economics.

## 4. Throughput Analysis and Energy Efficiency Ratio

In this section, we analyze achievable throughput of the SU and then we aim at calculating optimal accessing time ($\Gamma_{s}^{*}$) to maximize achievable throughput of the SU.

**Lemma 1.**  *$C_{s}\left(\Gamma_{s}\right)$ is concave while $0\leq \Gamma_{s}\leq 1$*.

**Proof.**  Obviously, $C_{s}=\Gamma_{s}R_{s}$ is a continuous function when $0\leq \Gamma_{s}\leq 1$. Thus, we discuss the second derivative of SU’s achievable throughput to demonstrate its concavity.

The first derivative of $C_{s}=\Gamma_{s}R_{s}$ with respect to $\Gamma_{s}$ can be expressed as

(6) $$\begin{array}{cc} \frac{\partial C_{s}}{\partial \Gamma_{s}} & =\frac{\partial (\Gamma_{s}R_{s})}{\partial \Gamma_{s}}=R_{s}+\Gamma_{s}\frac{\partial R_{s}}{\partial \Gamma_{s}}\\ & =\log_{2}\left(1+\frac{h(1-\Gamma_{s})X}{\Gamma_{s}N_{0}}\right)-\frac{1}{\ln2}\frac{1}{\Gamma_{s}+\frac{h(1-\Gamma_{s})X}{N_{0}}}\frac{hX}{N_{0}} \end{array}$$

Then the second derivative of $C_{s}=\Gamma_{s}R_{s}$ with respect to $\Gamma_{s}$ can be further obtained

(7) $$\begin{array}{cc} \frac{\partial^{2}C_{s}}{\partial \Gamma_{s}^{2}} & =-\frac{1}{\ln2}\frac{\frac{hX}{N_{0}}}{\left(\Gamma_{s}+\frac{(1-\Gamma_{s})hX}{N_{0}}\right)\Gamma_{s}}+\frac{1}{\ln2}\frac{\frac{hX}{N_{0}}\left(1-\frac{hX}{N_{0}}\right)}{{\left(\Gamma_{s}+\frac{(1-\Gamma_{s})hX}{N_{0}}\right)}^{2}}\\ & =\frac{1}{\ln2}\frac{-\frac{{\left(hX\right)}^{2}}{N_{0}^{2}}}{{\left(\Gamma_{s}+\frac{(1-\Gamma_{s})hX}{N_{0}}\right)}^{2}\Gamma_{s}} \end{array}$$

Thus, for X>0 and $0\leq \Gamma_{s}\leq 1$, we have

(8) $$\frac{\partial^{2}C_{s}}{\partial \Gamma_{s}^{2}}<0$$

which proves the concavity of SU’s achievable throughput for $0\leq \Gamma_{s}\leq 1$.

**Theorem 1.**  *$C_{s}\left(\Gamma_{s}\right)$ reaches the global optimality at*

(9) $$\Gamma_{s}^{*}=\frac{W(\frac{\frac{hX}{N_{0}}-1}{e})}{\left(W\left(\frac{\frac{hX}{N_{0}}-1}{e}\right)+1\right)\left(\frac{hX}{N_{0}}-1\right)}$$

*where W(·) refers to the Lambert W function*.

**Proof.**  We denote the stationary point of SU’s achievable throughput by $\Gamma_{s}$ and consequently we have

$$\frac{\partial C_{s}}{\partial \Gamma_{s}}{|}_{\Gamma_{s}=\Gamma_{s}^{*}}=0.$$

From Lemma 1, It can be inferred that $C_{s}\left(\Gamma_{s}\right)$ reaches the global optimality at $\Gamma_{s}$ for $0\leq \Gamma_{s}\leq 1$.

We define

$$L=\frac{\Gamma_{s}\left(\frac{hX}{N_{0}}-1\right)}{\Gamma_{s}+\left(1-\Gamma_{s}\right)\frac{hX}{N_{0}}}.$$

Then $\Gamma_{s}$ can be derived as follows:

(10) $$\log_{2}\left(1+\frac{(1-\Gamma_{s})hX}{\Gamma_{s}N_{0}}\right)-\frac{1}{\ln2}\frac{\frac{hX}{N_{0}}}{\Gamma_{s}+\left(1-\Gamma_{s}\right)\frac{hX}{N_{0}}}=0$$

(11) $$\Rightarrow \ln\left(L\right)+\ln\left(\frac{hX}{N_{0}}-1\right)=L+1$$

(12) $$\Rightarrow \Gamma_{s}=\frac{W(\frac{\frac{hX}{N_{0}}-1}{e})}{\left(W\left(\frac{\frac{hX}{N_{0}}-1}{e}\right)+1\right)\left(\frac{hX}{N_{0}}-1\right)}$$

**Figure 5:**
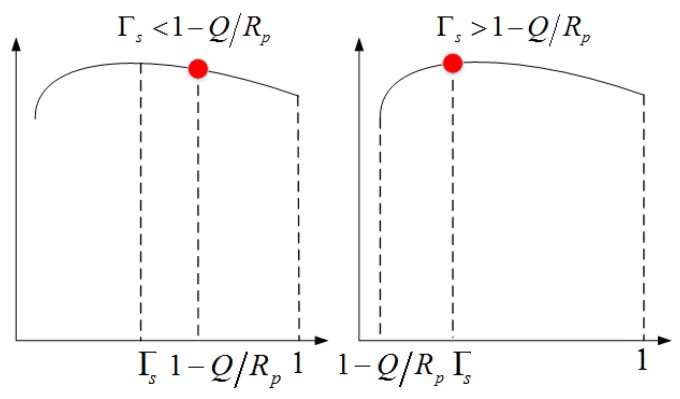
Graphical interpretation for the optimal save-ratio in leasing and energy harvesting transmission mode.

Since $\Gamma_{s}$ is bounded within $\left[0,1-\frac{Q}{R_{p}}\right]$, in which $C_{s}\left(\Gamma_{s}\right)$ is concave according to Lemma 1, the optimal accessing time $\Gamma_{s}^{*}$ of SU can be derived as

(13) $$\Gamma_{s}^{*}=min\left\{1-Q/R_{p},\Gamma_{s}\right\}$$

which is graphically illuminated by Figure 5 (where the red dot represents the optimal leasing time).

According to solving the optimal $\Gamma_{s}^{*}$, we can calculate the achievable throughput of the primary user $C_{p}$ as follows,

(14) $$C_{p}=\Gamma_{p}R_{p}=\left(1-\Gamma_{s}^{*}\right)\log\left(1+SNR_{p}\right)$$

where $SNR_{p}$ is signal noise ratio $SNR_{p}=\frac{P_{p}}{N_{0}}$ and $P_{p}$ is the power of PU transmitter and $N_{0}$ is additive white gaussian noise.

Based on the above analysis, we can obtain energy efficiency ratio *η* of the whole network, which is the ratio between the achievable utility and the consumption of energy, as follows:

(15) $$\eta =\left(U_{p}+U_{s}\right)/E_{p}$$

## 5. Numerical Results and Analysis

In this section, numerical simulations are conducted to evaluate the performance of the proposed framework. We consider the energy harvesting based spectrum leasing scheme in cognitive radio network with one PU and one SU. The PU could lease a part of spectrum to the SU. Additional white gaussian noise $N_{0}$ = −60 dbm, channel gain *h* equals to 0.75, the equivalent revenue per unit satisfactory ωp is set to 1.5, payment of unit time *c* is 1.1, satisfactory factor of the PU *α* = 0.8, satisfactory factor of the SU b is set to 0.6, the min traffic data requirement of PU *Q* is 30 bit.

**Figure 6:**
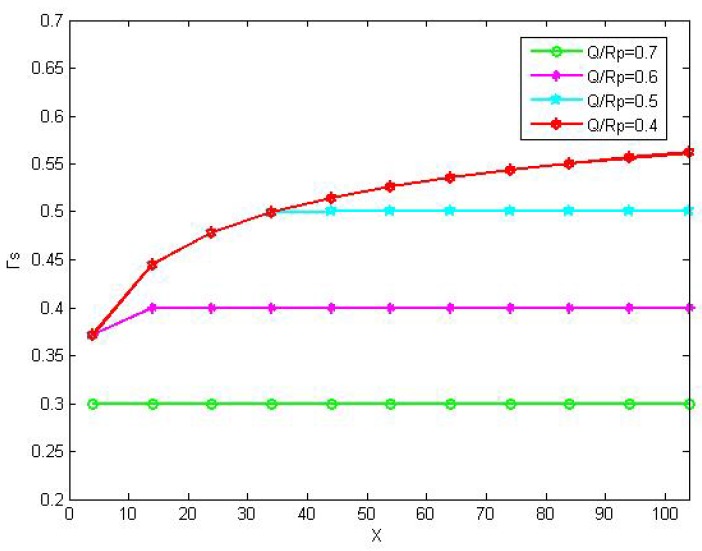
Optimal save-ratio in leasing and energy harvesting mode with different $\frac{Q}{R_{p}}$.

In Figure 6, we evaluate the optimal access time $\Gamma_{s}^{*}$ *versus* the energy harvesting rate *X* with different $Q/R_{p}$. It can be observed in this figure that the the optimal access time $\Gamma_{s}^{*}$ increases as *X* does. But it can not exceed the value $1-Q/R_{p}$. This is because the energy can only be harvested from signal of the PU and the frame is constant. With the increasing of $\Gamma_{s}$, there is less time left for the energy harvesting, and the transmission power of SU will decrease while its transmission time increases. On the contrary, a small $\Gamma_{s}$ will result in a greater transmission power and longer transmission time of SU. Therefore, we should find out the optimal access time to maximize utility of the entire network.

**Figure 7:**
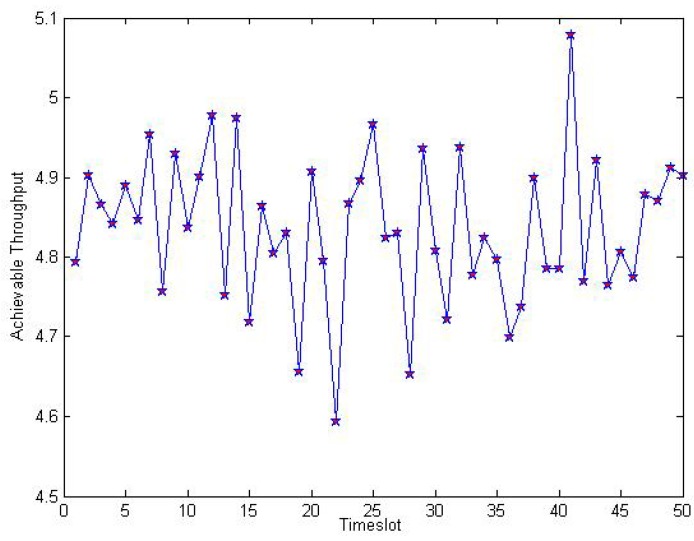
Achievable throughput trace with X∼Γ(10,5) and $h∼Exp(\frac{1}{500})$.

Figure 7 illustrates achievable throughput trace with X∼Γ(10,5) with respect to the different timeslot in the dynamic spectrum leasing scheme. Due to the randomness of energy harvesting rate, it has a great impact on $\Gamma_{s}$, so the achievable throughput fluctuates randomly, but its value keeps within a level around constant value 4.8.

**Figure 8:**
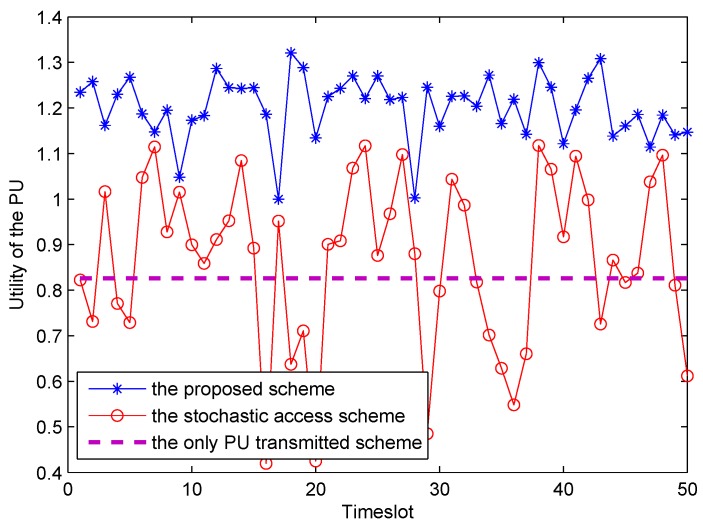
Utility of the PU in the different scheme.

Figure 8 shows the utility of the PU with respect to different *X* in the corresponding time-slot for different access schemes. As shown in Figure 8, it is obvious that utility of our proposed spectrum leasing scheme outperforms that of the stochastic access scheme to a large extent. In the stochastic access scheme, the $\Gamma_{s}$ follows a Poisson distribution. Due to the monopolizing on the entire frame by PU in the only PU transmitted scheme, the energy harvesting ratio is not relevant to utility of the PU. Therefore, the utility of PU is identically equals to 0.826. In conclusion, this is the reason why the PU is willing to rent a fraction of time slot to the SU.

**Figure 9:**
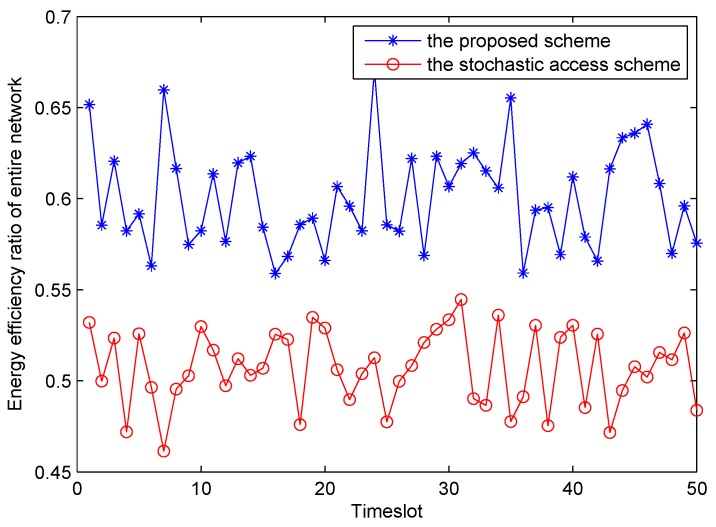
The energy efficiency ratio *η* in the different scheme.

We also compared the energy efficiency ratio of our proposed energy harvesting mode based on optimal leasing time with that of stochastic access. The simulation results (50 time-slot energy efficiency ratio traces) with different probability distributions of energy harvesting ratio *X* are depicted in Figure 9, apparently, it can be seen that energy utilization rate of our proposed scheme is higher than that of stochastic scheme. Overall, obvious superiority and viability of the proposed scheme in this paper have been confirmed.

## 6. Conclusions

In this paper, we propose a spectrum leasing framework in cognitive radio network with energy harvesting. The system consists of one primary user and one secondary user, both of them transmit in the model of time-slot. In each frame, the PU could allocate a portion of its transmission time to SU. When the PU transmits its own data on the licensed channel owned by PU, the SU could harvest energy from the radio signal of the PU. The SU is powered exclusively by the harvested energy. The SU can transmit its data by making a payment for PU. We optimize save-ratio, transmission duration while keeping PU sufficiently protected. Through analyzing, we obtain the optimal the transmission time slot of SU so that maximize throughput of SU. Then we also calculated EER of the whole network. Finally, numerical simulation results prove the proposed scheme significantly outperforms the other schemes.

In our future research, we will jointly consider multiple SUs selection and channels assignment to enhance spectrum efficiency. Moreover, we intend to extend our current investigation to the problem of selecting multiple SUs for each primary transmitter.
